# Dried Blood Spot Biomarkers of Oxidative Stress and Inflammation Associated with Blood Pressure in Rural Senegalese Women with Incident Hypertension

**DOI:** 10.3390/antiox10122026

**Published:** 2021-12-20

**Authors:** Yan Lin, Xiangtian Wang, Luciane Lenz, Ousmane Ndiaye, Jian Qin, Xiaoli Wang, Hui Huang, Marc A. Jeuland, Junfeng Zhang

**Affiliations:** 1 Nicholas School of the Environment & Duke Global Health Institute, Duke University, Durham, NC 27705, USA; yan.lin@duke.edu (Y.L.); xiangtian.wang@duke.edu (X.W.); qinjian@gxmu.edu.cn (J.Q.); tjutwxl@163.com (X.W.); hhlsk@126.com (H.H.); 2 RWI Leibniz Institute for Economic Research, 10115 Berlin, Germany; luciane.lenz@rwi-essen.de (L.L.); marc.jeuland@duke.edu (M.A.J.); 3 Centre de Recherche pour le Développement Economique et Social (CRDES), Université Gaston-Berger, Saint-Louis, P.O. Box 234, Senegal; ousmanendiayeptci@gmail.com; 4 School of Public Health, Guangxi Medical University, Nanning 530021, China; 5 School of Environmental Science and Safety Engineering, Tianjin University of Technology, Tianjin 300387, China; 6 College of Public Health, Zhengzhou University, Zhengzhou 450001, China; 7 Sanford School of Public Policy and Duke Global Health Institute, Duke University, Durham, NC 27705, USA

**Keywords:** dried blood spot, blood pressure, hypertension, c-reactive protein, malondialdehyde, biomarker

## Abstract

Blood biomarkers of oxidative stress and inflammation have been associated with increased risk of hypertension development; yet their application in sub-Saharan Africa has been limited due to the lack of blood collection facilities. In this study, we evaluated the usefulness of dried blood spots (DBS), a more feasible alternative to venous blood, in rural sub-Saharan residents. We recruited 342 women with incident hypertension from rural Senegal, and measured C-reactive protein (CRP) and malondialdehyde (MDA) in DBS and concurrent blood pressure (BP) at baseline and 1-year follow-up. Associations of DBS biomarkers with current levels of and 1-year changes in BP were examined after adjusting for demographic, medical, and socioeconomic covariates. DBS concentrations of MDA were significantly associated with concurrent systolic BP (SBP) (*p* < 0.05), while DBS baseline concentrations of CRP were associated with longitudinal changes in SBP between baseline and follow-up. Compared to participants with baseline CRP < 1 mg/L, those with CRP of 1–3 mg/L and 3–10 mg/L had 2.11 mmHg (95%CI: −2.79 to 7.02 mmHg) and 4.68 mmHg (95%CI: 0.01 to 9.36 mmHg) increases in SBP at follow-up, respectively. The results support the use of DBS biomarkers for hypertension prevention and control, especially in settings with limited clinical resources.

## 1. Introduction

Hypertension is a leading modifiable cardiovascular risk factor worldwide [1]. From 1975 to 2015, the mean systolic and diastolic blood pressures increased rapidly in sub-Saharan African populations, even as the same measures decreased in most high-income countries [2]. Consequentially, the average blood pressure (BP) levels in sub-Saharan African populations are now among the highest in the world [2]. The inadequate diagnosis and control of high BP in sub-Saharan African countries has contributed to a rising burden of cardiovascular diseases, which have now become the leading cause of death among adults over the age of 30 years in the region [3]. Due to the high cost of management of cardiovascular complications, and the lack of access of many people to such services, the prevention and control of hypertension is crucial to reduce public health burdens and costs in sub-Saharan Africa. It is critically important in the region to recognize environmental risk factors, understand the mechanisms underlying the development and progression of hypertension, as well as identify individuals with higher susceptibility and risks.

While the pathogenesis of hypertension is complicated and involves multiple mechanisms, systemic inflammation and oxidative stress have been shown to closely track increased BP. A number of cross-sectional studies have shown positive associations of increased BP with measures of C-reactive protein (CRP) and malondialdehyde (MDA) in blood [4,5]. Prospective studies have further shown that baseline levels of CRP are significantly associated with the incidence of hypertension among normotensive individuals [6,7], and with hypertension remission among hypertensive patients [8]. On the other hand, CRP and MDA have been shown to change in response to environmental exposures, such as air pollution [9,10] and diets [11,12], making them useful biomarkers for identifying environmental risk factors of cardiovascular diseases. Their applicability in large population studies in resource-limited settings such as sub-African countries has, however, often been restricted by the challenges of invasive venous blood collection and subsequent specimen processing and storage. In particular, trained phlebotomists or conveniently accessed facilities where the collected blood can be readily processed are difficult to find in all but the largest urban centers in the region.

Drops of whole blood can be self-collected via finger pricking, blotting, and drying on filter papers as dried blood spots (DBS). Studies have shown that people are generally more willing to provide DBS than venous blood samples [13]. DBS have proven over the past few decades to be promising specimens that can be analyzed to provide a variety of health or disease indicators. For example, a growing number of DBS biomarkers, such as CRP and MDA, have been developed to track inflammation and oxidative stress [14]. These specific DBS biomarkers may be particularly useful in resource-limited areas of sub-Saharan Africa where they can assist in hypertension prevention and control. Despite the promise, information remains scarce on the relationship between DBS biomarkers and BP in sub-Saharan Africans.

In a recent longitudinal study in rural Senegal, we evaluated the effects of household air pollution from different types of improved cooking stoves on several health indicators (e.g., respiratory symptoms and BP) in women primary cooks. We also collected DBS from these women. In the present analysis, we aim to examine the relationship between BP measures and DBS CRP and MDA levels, including whether baseline levels of CRP or MDA were associated with changes in BP observed over a 1-year period.

## 2. Method

### 2.1. Study Participants

The present analysis leverages an existing cohort from rural Senegal, which was primarily established to evaluate the effects of different types of improved cooking stoves on a range of outcomes including fuel use, household air pollution levels, and associated health outcomes. We recruited participants from 15 rural villages located in six districts of Saint-Louis in northern Senegal and Kaffrine in central Senegal. In each village, households were randomly selected using a field-based counting method. Specific individuals within those households were recruited as follows. All study participants were women who identified as cooks and were exposed to household air pollution. Given that many households in Senegal contain multiple cooks who sometimes share or rotate responsibility for this task, at baseline, the woman who identified as the primary cook was enrolled into the study. In cases where this primary cook was not available, a secondary cook was enrolled instead. Monitoring procedures were non-invasive, and no women were excluded based on specific criteria. The monitoring procedures included self-reported health symptoms and diagnosis history, blood pressure and oxygen measurements, exposure assessments among a randomly assigned subset of women, and DBS collection. All women enrolled in the study were asked to give informed consent for each monitoring procedure, and nearly all agreed to full participation.

Following enrollment and baseline surveys, each household was randomly assigned to one of three groups: a control group maintained their traditional cooking stove while two treatment groups received one of two improved cooking stoves. For each participant, trained enumerators coordinated two on-site visits in March and April of 2018 and 2019, to measure BP, collect DBS samples, and conduct a detailed review of recent respiratory symptoms and exposures. With a finger prick using a disposable safety lancing device, four DBS samples were collected with a Whatman 903 Protein Saver card, dried at room temperature, stored in a freezer, and shipped to Duke University for laboratory analysis. All measurements were taken by certified Senegalese nurses with additional training on safety and hygiene regulations. This study was approved by the institutional review board of Duke University, and was conducted with the support of the Ministry of Energy in Senegal.

In the current analysis, we only included participants with incident hypertension, according to at least one of the following three criteria: (1) reported prior diagnosis of hypertension; (2) baseline systolic BP (SBP) ≥ 130 mmHg; or (3) baseline diastolic BP (DBP) ≥ 80 mmHg [15]. Of note, participants who met the latter two criteria were informed of their potential hypertensive status, but no medical advice was provided.

### 2.2. BP Measurement

Peripheral BP was measured during each visit by a trained nurse. Participants were asked to sit in a quiet room with a comfortable temperature, position their arm on a flat surface at a level even with the heart, and to relax for a few minutes. Three measurements of SBP and DBP were recorded at intervals of 1–2 min, to ensure consistency, using an Omron M6 Upper Arm Blood Pressure Monitor with the standard upper arm cuff size. Though we did not give instructions regarding diet and exercise prior to the blood pressure measurements, none of the women were smokers, and exercise among this population is uncommon, though many were involved in physical labor associated with farming or typical domestic tasks.

### 2.3. DBS Analysis

We extracted analytes from the DBS samples in a laboratory at Duke University as previously described [14]. Briefly, two pieces of each DBS sample, punched with a 3 mm Miltex disposable biopsy punch, were mixed with 150 μL phosphate buffered saline buffer containing 0.1% Triton X100 and 0.03% Tween-20. The mixture was incubated on a shaker for 30 min, transferred to a Spin-X tube, and centrifuged at 8000 g for 10 min for the collection of blood elute. An additional wash was performed with 50 μL buffers and the final elute volume was 200 μL.

We used a previously established method to determine MDA levels in DBS samples [14]. Briefly, 20 µL DBS extract was incubated with 65 µL 1N NaOH in a 1.5 mL screw cap tube at 60 °C for 30 min. Subsequently we added 65 µL 1N HCl, 750 µL phosphoric acid, and 150 µL TBA solution to each vial, and incubated the mixture at 80 °C for 1 h. The solution was then cooled to room temperature and injected into a Waters 2695 HPLC system with a multi λ fluorescence detector (Waters 2475). We used a Hypersil Gold column (C8, 5 µm, 4.6 × 150 mm, Waters) for chromatographic separation. The excitation and emission wavelength lengths were 532 nm and 553 nm, respectively. The level of CRP in DBS extract was measured with a commercial ELISA kit (The Cayman Chemical). Concentrations of both CRP and MDA were normalized by hemoglobin levels of the DBS extract, which was measured with spectrophotometry at a wavelength of 410 nm [14]. The reproducibility of this method was evaluated by analyzing 40 DBS samples collected from the same finger prick obtained from 10 people as described previously [14]. The coefficient of variation is 11.2% for CRP and 23.1% for MDA. We used an empirical equation derived from our previous cross-validation study to transform the DBS CRP levels to plasma levels, which were used for risk scale calculation [14].

### 2.4. Statistical Analysis

The normality of each biomarker was examined with a Kolmogorov–Smirnov test. We tabulated the mean ± standard deviations (SD) or geometric mean (interquartile range, IQR) as appropriate. We first compared the levels of BP and DBS biomarkers at baseline and follow-up using mixed effects models with random intercepts for each study participant. We then tested the correlation of outcomes between baseline and follow-up using a Spearman correlation test. Next, we examined the associations between levels of BP and DBS biomarkers using mixed effects models controlling for random participant intercepts, and fixed effects for time-varying covariates including secondhand smoke exposure, respiratory symptoms (cough, chest tightness, and breath difficulty) in the past 2 weeks, time of day of the measurement, and the DBS biomarker type (CRP or MDA). Lastly, we studied the effects of baseline levels of DBS biomarkers on 1-year changes in BP. In this analysis, we modeled the change of BP as a function of CRP relative risk scale or quartiles of DBS biomarkers as categorical variables (Model 1), with adjustment for demographic and medical (i.e., ethnic group, age, self-reported diagnosis of hypertension, history of asthma and cardiovascular diseases, Model 2), and socioeconomic covariates (i.e., villages, education levels, and household stove types, Model 3). Linear trends across increasing risk scales of CRP or quartiles of DBS biomarkers were tested using models in which BP changes were examined as a function of biomarker risk scales or quartiles as ordinal variables. Alpha was set at 0.05, and all tests were two-tailed. All data analyses were performed in statistical software R (version 3.3.2).

## 3. Results

### 3.1. Demographics of Study Participants

**Figure 1:**
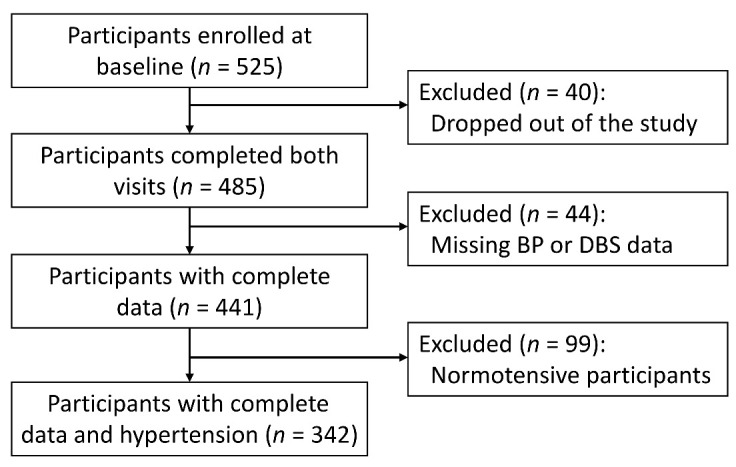
Flow chart of the analytical sample.

Of the 525 participants enrolled at baseline, 40 and 44 participants dropped out of the study or missed at least one BP measurement or DBS collection, respectively, resulting in 441 participants with complete data (Figure 1). Of the 441 participants, 275 had baseline SBP ≥ 130 mmHg or DBP ≥ 80 mmHg, while 67 had normal BP levels but self-reported a prior diagnosis of hypertension. These 342 hypertensive participants were included in the current analysis (Figure 1).

The study population was evenly distributed across districts (Table 1). The mean ( ± SD) age of the 342 participants at baseline was 33 ± 10 years, and most of them are of Poulard or Wolof ethnicity with educational levels below middle school (Table 1). The average SBP and DBP was 126 ± 19 and 86 ± 12 mmHg, respectively. Compared with the excluded 99 normotensive participants, the study population had significantly higher levels of SBP and DBP, while the levels of pulse pressure, CRP and MDA were comparable (Table 1).

### 3.2. BP and DBS Biomarkers at Baseline and Follow-Up

Compared with the baseline values, participants’ SBP, DBP, and pulse pressure significantly decreased over the timeframe of the study, by 9.4%, 9.1%, and 9.9%, respectively; MDA levels in DBS decreased by 42% (95%CI: 35% to 50%); but CRP levels increased by 30% (95%CI: 12% to 51%) (Table 2). There were significant positive correlations between baseline and follow-up levels of SBP, DBP, pulse pressure, and CRP (*p* < 0.001, Table 2). However, MDA levels measured at baseline and follow-up were not significantly correlated (*p* = 0.34).

### 3.3. Associations between BP and DBS Biomarkers

**Figure 2:**
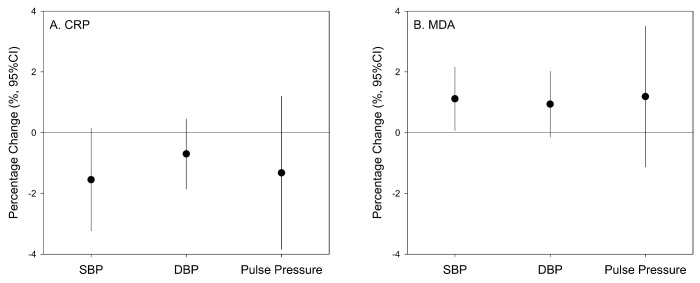
Percentage changes of blood pressure measures associated with a 1-SD increment in the natural logarithm of CRP (1.30, panel **A**) and MDA (0.89, panel **B**) concentrations in DBS samples. Associations were tested using linear mixed effects models with random intercepts for study participants, adjusted for the fixed effects of secondhand smoke exposures, respiratory symptoms (cough, chest tightness, and breath difficulty) in the past 2 weeks, time of the day, and the other DBS biomarker (CRP or MDA).

We used mixed effects models with random intercepts of participants and fixed effects of known time-varying covariates to examine the associations between BP and DBS biomarkers (Figure 2). We found that a 1-SD increment in the natural logarithm of MDA levels (0.89) was significantly associated with a 1.11% (95% CI: 0.07% to 2.15%) increase in SBP, but not DBP or pulse pressure (Figure 1). In contrast, we observed no associations between CRP concentration and any BP measures (Figure 2).

### 3.4. Associations between Baseline DBS Biomarkers and 1-Year BP Changes

We categorized study participants according to baseline CRP risk scales or quartiles of DBS biomarkers, and examined the effects of categorized biomarker levels on participants’ 1-year BP changes (ΔBP, Table 3). Compared with participants with CRP levels below 1 mg/mL, the crude model shows increase in ΔSBP levels of 2.57 mmHg (95% CI: −2.13 to 7.27 mmHg) and 5.78 mmHg (95%CI: 1.24 to 10.3 mmHg) among participants with CRP levels of 1–3 mg/L and 3–10 mg/L, respectively (Table 3). The trend across the three CRP risk scales and the ΔSBP elevation at CRP levels of 3–10 mg/L reached statistical significance (*p* < 0.05), which remained robust after adjusting for demographic and medical (Model 2) and socioeconomic (Model 3) covariates (Table 3). We did not observe significant increases in ΔSBP among participants with CRP levels above 10 mg/L, which implies a possible influence of acute phase responses [16]. We found no significant effects of CRP risk scales on changes of DBP or pulse pressure. Similarly, there were no effects of CRP or MDA quartiles on any BP measure (Tables S1 and S2).

## 4. Discussion

Hypertension is a leading cardiovascular risk factor in sub-Saharan Africa. Understanding the mechanisms and risk factors contributing to elevated BP in this region is crucial for the prevention and control of hypertension. In this study, we used DBS biomarker levels, which can be readily assessed in large populations at low collection cost, to examine the relationship of inflammation and oxidative stress with current BP levels and BP longitudinal changes among hypertensive women residing in rural Senegal. We found that CRP levels were associated with longitudinal SBP changes, whereas MDA levels were associated with concurrent SBP levels. These results suggest that increased inflammation and oxidative stress may contribute to elevated SBP in this population and support the use of DBS specimens for monitoring BP risk indicators.

CRP levels have been frequently shown, independent of multiple risk factors, to predict the onset of hypertension [6,7] as well as future increases in SBP but not DBP [17] among normotensive individuals. In animal models, overexpressing CRP has caused elevated SBP, likely due to CRP-induced endothelial dysfunction and oxidative stress, which further supports a causal interpretation of the effect of CRP on elevated BP [18,19]. Among hypertensive individuals, CRP levels have been found to be positively associated with arterial stiffness [20] and end-organ damage [21], and have been shown to predict future cardiovascular events [5] and the rate of hypertension remission [8]. We found in this study that higher baseline CRP levels in DBS specimens were associated with SBP increases over 1 year follow-up in a dose-dependent manner among hypertensive women with a CRP level below the cut-off value of acute-phase inflammation (i.e., 10 mg/L) [16]. To the best of our knowledge, this is the first prospective study of the relationship between CRP and BP in sub-Saharan Africa where only a few case-control and cross-sectional studies have previously found higher blood CRP levels associated with increased BP [22,23]. In North America and Europe, a small number of prospective studies have investigated CRP alongside BP trajectories [17]. Venous blood samples were used in these studies to measure CRP, which is impractical in a study setting like ours. Given the substantial differences in CRP levels and its predictive values across countries and ethnicities as documented in previous studies [24], our results add new evidence from sub-Saharan African villages, and suggested the use of DBS in resource-limited settings as an alternative biospecimen to the venous blood.

Thresholds have been reported for the cardiovascular effect of CRP in vitro [25], while a nonlinear relationship between CRP levels and future cardiovascular events was observed in previous population studies [5]. Consistent with these findings, we observed a significant association between baseline CRP risk cut-off categories and 1-year change in SBP. It is important to note that the risk cut-off values we used were based on studies mostly conducted in North America and Europe [26]. Studies in other regions have suggested different cut-off values, likely due to differences in race, diet, and/or various cardiovascular and metabolic risk factors [27]. Among the female participants in rural Senegal included in this study, a large proportion (24.5%) of baseline samples indicate likely acute-phase inflammatory responses with CRP levels exceeding 10 mg/L [16], which were not associated with BP changes. Therefore, the identification of participants whose CRP levels were influenced by infection or other acute conditions could be pivotal to better using DBS CRP to identify individuals with increased cardiovascular risk in this region.

Oxidative stress has also been suggested to play an essential role in hypertension pathophysiology due to its destructive effects on the vascular wall, kidneys, and central nervous system [28]. In our study, we observed significant decreases in BP at follow-up, that were associated with decreased MDA levels in DBS, while the levels of CRP had increased at follow-up. These results suggest a possible linkage between levels of oxidative stress and BP among the study participants. However, baseline MDA levels were not associated with BP changes over time. A possible explanation is that DBS MDA is not a good biomarker for long-term oxidative stress in the studied population given poor correlations of MDA between baseline and follow-up (Table 2). In contrast, CRP is considered to reflect chronic inflammation and exhibits a lack of diurnal sensitivity or age dependence [16]. Indeed, baseline and follow-up CRP levels were significantly correlated in our study.

Our results indicated that DBS CRP levels, as indicators of chronic inflammation, were associated with SBP trajectory, while short-term variations in DBS MDA levels were associated with concurrent SBP levels. Of note, both systemic inflammation and oxidative stress have been shown to perturb nitric oxide synthesis and cause endothelial dysfunction [25,28], which may play vital roles in the development of hypertension [29]. Thus, future efforts to develop DBS biomarkers for endothelial dysfunction would be useful to better understand the pathophysiology of hypertension in resource-limited settings. On the other hand, all the study participants are primary cooks routinely exposed to household air pollution, and recent meta-analyses have shown that CRP levels had stronger associations with long-term as opposed to short-term exposures to air pollution [30], while MDA levels were associated with short-term exposures [31]. Taken together, these results highlight the importance of identifying environmental exposures contributing to increased chronic inflammation and acute oxidative stress, which may inform development of future strategies for hypertension prevention and control.

### Study Limitations

The present study leveraged an existing longitudinal study in rural Senegal that aimed to evaluate the health effects of household air pollution. As the cohort was not originally designed to examine the relationship between DBS biomarkers and BP, the present analysis has several limitations. First, we did not assess several factors that may confound the relationship between DBS biomarkers and BP, such as lifestyle, anti-hypertensive medication, psychosocial stress, obesity, and metabolic syndrome. Additionally, we did not measure other conventional cardiovascular risk factors such as cholesterol levels. Second, while this study focused on participants with incident hypertension, the hypertension status was not among the inclusion criteria applied during participant recruitment. Instead, we retrospectively identified hypertensive individuals based on self-reported diagnosis of hypertension and baseline BP measurements. Third, the participants may have changed their lifestyle and behaviors during the 1 year follow up. For example, many participants used improved cookstoves after the baseline survey and measurements—and those with high BP were informed of their potential hypertensive status at the same time. Both of these may contribute to the reduced BP levels observed at follow-up, though we did not observe relationships between assignment to the improved cookstove intervention and BP or the DBS biomarkers. Nevertheless, we have accounted for these factors in statistical models by controlling for indicator variables of household cookstove types at follow-up, as well as participants’ self-consciousness of hypertensive status at baseline. Lastly, the statistical analysis on the association between DBS CRP levels and 1-year BP changes may be underpowered, because the sample size of the original study was calculated to provide sufficient power only to detect the effects of the original cooking stove intervention on longitudinal changes in health outcomes. This partially explains why significant effects of CRP were observed only with high-risk (i.e., 3–10 mg/L) but not moderate-risk (i.e., 1–3 mg/mL) scales.

## 5. Conclusions

Among women with incident hypertension living in rural Senegal, DBS CRP, a biomarker of chronic inflammation, was significantly associated with SBP change over a 1-year period; while DBS MDA, a biomarker of acute oxidative stress, was significantly associated with concurrent SBP levels. These results provide associative evidence from a rural sub-Saharan setting linking increasing levels of DBS biomarkers to worsened BP levels or trajectory. While the confounding effects of several factors cannot be ruled out in the study, our findings on DBS biomarkers are consistent with previous studies based on venous blood analysis. These findings thus support further application of these DBS biomarkers in future population studies to identify environmental and socio-behavioral factors causing increased levels of CRP and MDA, which may help with hypertension prevention and management. In addition, future efforts to develop additional DBS biomarkers reflecting cardiovascular health risks related to oxidative stress and systemic inflammation (e.g., endothelial dysfunction and cardiometabolic dysfunction) are also recommended.

## Figures and Tables

**Table 1:** Baseline characteristics of hypertensive and normotensive (excluded) participants.

Characteristic	Hypertensive (*n* = 342) ^a^	Normotensive (*n* = 99)	*p*-Value ^b^
Age, years	33 ± 10	28 ± 9	<0.001
Ethnicity			0.84
Poulard	82 (24%)	21 (21%)	
Wolof	246 (72%)	76 (77%)	
Others	14 (4%)	2 (2%)	
Education			0.002
Religious school	96 (28%)	37 (37%)	
< Middle school	234 (68%)	55 (56%)	
Middle or high school	12 (4%)	4 (4%)	
> High school	0 (0%)	3 (3%)	
District			0.40
Birkelane (in Kaffrine)	22 (6%)	8 (8%)	
Kaffrine (in Kaffrine)	67 (20%)	24 (24%)	
Koungheul (in Kaffrine)	60 (18%)	21 (21%)	
Dagana (in Saint Louis)	92 (27%)	27 (27%)	
Podor (in Saint Louis)	49 (14%)	11 (11%)	
Saint Louis (in Saint Louis)	52 (15%)	8 (8%)	
Systolic blood pressure, mmHg	126 ± 19	112 ± 9	<0.001
Diastolic blood pressure, mmHg	86 ± 12	72 ± 5	<0.001
Pulse pressure, mmHg	40 ± 14	39 ± 9	0.53
C-reactive protein, µg/g hemoglobin	2.1 (1.0–5.0)	2.2 (0.7–5.9)	0.80
Malondialdehyde, µg/g hemoglobin	103 (91–155)	112 (89–153)	0.41

a. Data are mean ± standard deviations, *n* (%), or geometric mean (interquartile range); b. Between-group differences are tested by one-way ANOVA or chi-square test.

**Table 2:** Blood pressure and DBS biomarker at baseline and follow-up.

Parameter	Visit 2018	Visit 2019	Percentage Change (95%CI) ^b^	Spearman’s Q
Systolic blood pressure, mmHg	126 ± 19 ^a^	115 ± 17	−9.4 (−10.8, −8.0)	0.54 ^c^
Diastolic blood pressure, mmHg	86 ± 12	78 ± 12	−9.1 (−10.7, −7.6)	0.42 ^c^
Pulse pressure, mmHg	40 ± 14	36 ± 11	−9.9 (−13.3, −6.5)	0.42 ^c^
C-reactive protein, µg/g hemoglobin	2.1 (1.0, 5.0)	2.8 (1.1, 7.6)	30 (12, 51)	0.41 ^c^
Malondialdehyde, µg/g hemoglobin	104 (91, 155)	59 (45, 83)	−42 (−50, −35)	0.05

a. Data are mean ± standard deviations or geometric mean (interquartile range); b. Estimated by mixed-effects models with random intercepts for study participants; c. Significant correlations between data at baseline and follow-up (*p* < 0.001).

**Table 3:** Relative changes of blood pressure by baseline CRP levels.

Parameters	CRP Values				P_trend_ Value (CRP < 10 mg/L)	P_trend_ Value (All Ranges)
	<1 mg/L (*n* = 116)	1–3 mg/L (*n* = 79)	3–10 mg/L (*n* = 89)	>10 mg/L (*n* = 85)		
**Relative ΔSBP (95% CI, mmHg)**						
Model 1 ^a^	0.00 (ref)	2.57 (−2.13, 7.27)	5.78 (1.24, 10.3)	2.23 (−2.95, 7.41)	0.01	0.10
Model 2 ^b^	0.00 (ref)	2.06 (−2.78, 6.89)	4.98 (0.38, 9.58)	1.66 (−3.53, 6.85)	0.03	0.19
Model 3 ^c^	0.00 (ref)	2.11 (−2.79, 7.02)	4.68 (0.01, 9.36)	2.01 (−3.27, 7.30)	0.04	0.18
**Relative ΔDBP (95% CI, mmHg)**						
Model 1	0.00 (ref)	3.11 (−0.55, 6.77)	2.63 (−0.91, 6.16)	3.00 (−1.04, 7.04)	0.13	0.11
Model 2	0.00 (ref)	2.47 (−1.28, 6.22)	1.97 (−1.60, 5.53)	2.55 (−1.48, 6.57)	0.23	0.20
Model 3	0.00 (ref)	2.77 (−1.01, 6.54)	1.86 (−1.73, 5.46)	3.46 (−0.61, 7.53)	0.26	0.12
**Relative ΔPP (95% CI, mmHg)**						
Model 1	0.00 (ref)	0.54 (−3.14, 4.21)	−3.16 (−6.70, 0.39)	0.77 (−3.28, 4.82)	0.10	0.59
Model 2	0.00 (ref)	0.41 (−3.43, 4.26)	−3.01 (−6.67, 0.64)	0.88 (−3.24, 5.01)	0.12	0.68
Model 3	0.00 (ref)	0.65 (−3.22, 4.53)	−2.82 (−6.52, 0.87)	1.44 (−2.73, 5.62)	0.16	0.86

Abbreviations: CI: confidence interval; SBP: systolic blood pressure; DBP: diastolic blood pressure; PP: pulse pressure; CRP: C reactive protein. a: crude model; b: adjusted for ethnic, baseline age, self-reported hypertension diagnosis, and history of asthma and cardiovascular diseases; c: adjusted for the above plus household stove types, village, and education levels.

## Data Availability

The data presented in this study are available on request from the corresponding author.

## Supplementary Materials

The following are available online at https://www.mdpi.com/article/10.3390/antiox10122026/s1, Table S1: Relative changes of blood pressure by quartiles of baseline CRP levels; Table S2. Relative changes of blood pressure by quartiles of baseline MDA levels.
