# Supplementary material for: Dried Blood Spot Biomarkers of Oxidative Stress and Inflammation Associated with Blood Pressure in Rural Senegalese Women with Incident Hypertension
Source: Antioxidants (Basel). 2021 Dec 20;10(12):2026. doi: 10.3390/antiox10122026 (PMC8698702; doi:10.3390/antiox10122026)
Supplement: Supplementary file 1 [file antioxidants-10-02026-s001.zip › antioxidants-1500751-supplementary.pdf]

Supplementary Materials

Table S1. Relative changes of blood pressure by quartiles of baseline CRP levels.

Parameters	Quartiles of CRP Concentration (μg/g hemoglobin)				p_{trend} Value
	Q1 (< 0.98)	Q2 (0.98–2.05)	Q3 (2.05–5.03)	Q4 (>5.03)	
**Relative ΔsBP (95% CI, mmHg)**					
Model 1 [a]	0.00 (ref)	1.36 (−3.58, 6.30)	4.66 (−0.28, 9.60)	3.33 (−1.60, 8.25)	0.10
Model 2 [b]	0.00 (ref)	0.84 (−4.26, 5.94)	3.82 (−1.19, 8.84)	2.43 (−2.56, 7.42)	0.20
Model 3 [c]	0.00 (ref)	1.21 (−3.95, 6.38)	3.78 (−1.29, 8.84)	2.81 (−2.29, 7.91)	0.18
**Relative ΔdBP (95% CI, mmHg)**					
Model 1	0.00 (ref)	0.38 (−3.48, 4.23)	2.65 (−1.20, 6.50)	1.87 (−1.97, 5.71)	0.20
Model 2	0.00 (ref)	−0.63 (−4.58, 3.32)	1.60 (−2.28, 5.49)	1.04 (−2.82, 4.90)	0.39
Model 3	0.00 (ref)	−0.23 (−4.21, 3.74)	1.79 (−2.11, 5.68)	1.74 (−2.18, 5.67)	0.24
**Relative ΔPP (95% CI, mmHg)**					
Model 1	0.00 (ref)	−0.99 (−4.86, 2.89)	−2.01 (−5.89, 1.86)	−1.45 (−5.32, 2.41)	0.39
Model 2	0.00 (ref)	−1.47 (−5.54, 2.59)	−2.22 (−6.21, 1.77)	−1.39 (−5.36, 2.59)	0.45
Model 3	0.00 (ref)	−1.45 (−5.55, 2.65)	−1.99 (−6.01, 2.02)	−1.07 (−5.12, 2.97)	0.57

Abbreviations: CI - confidence interval; sBP - systolic blood pressure; dBP - diastolic blood pressure; PP - pulse pressure; CRP - C reactive protein. a. crude model; b. adjusted for ethic, baseline age, self-reported hypertension diagnosis, and history of asthma and cardiovascular diseases; c. adjusted for the above plus household stove types, village, and education levels.

Table S2. Relative changes of blood pressure by quartiles of baseline MDA levels.

Parameters	Quartiles of MDA concentration (μg/g hemoglobin)				p_{trend} value
	Q1 (< 91)	Q2 (91–117)	Q3 (117–155)	Q4 (>155)	
**Relative ΔsBP (95%CI, mmHg)**					
Model 1 [a]	0.00 (ref)	−1.94 (−6.88, 3.00)	−4.96 (−9.90, −0.02)	−1.34 (−6.26, 3.59)	0.38
Model 2 [b]	0.00 (ref)	−1.99 (−6.95, 2.98)	−4.26 (−9.21, 0.69)	−0.92 (−5.84, 3.99)	0.52
Model 3 [c]	0.00 (ref)	−2.08 (−7.15, 2.99)	−4.46 (−9.53, 0.62)	−1.02 (−5.99, 3.94)	0.49
**Relative ΔdBP (95%CI, mmHg)**					
Model 1	0.00 (ref)	−0.99 (−4.84, 2.85)	−3.30 (−7.15, 0.55)	−1.67 (−5.51, 2.16)	0.24
Model 2	0.00 (ref)	−1.11 (−4.95, 2.74)	−2.92 (−6.75, 0.92)	−1.31 (−5.12, 2.49)	0.35
Model 3	0.00 (ref)	−0.67 (−4.58, 3.24)	−2.30 (−6.21, 1.61)	−1.19 (−5.02, 2.64)	0.40
**Relative ΔPP (95%CI, mmHg)**					
Model 1	0.00 (ref)	0.95 (−2.93, 4.82)	1.66 (−2.21, 5.54)	−0.34 (−4.20, 3.53)	0.96
Model 2	0.00 (ref)	0.88 (−3.08, 4.84)	1.34 (−2.61, 5.29)	−0.39 (−4.31, 3.53)	0.91
Model 3	0.00 (ref)	1.41 (−2.61, 5.43)	2.16 (−1.87, 6.18)	−0.16 (−4.10, 3.77)	0.97

Abbreviations: CI - confidence interval; sBP - systolic blood pressure; dBP - diastolic blood pressure; PP - pulse pressure; MDA - malondialdehyde. a. crude model; b. adjusted for ethic, baseline age, self-reported hypertension diagnosis, and history of asthma and cardiovascular diseases; c. adjusted for the above plus household stove types, village, and education levels.
